# Improving the Clinical Interpretation of Transcutaneous Carbon Dioxide and Oxygen Measurements in the Neonatal Intensive Care Unit

**DOI:** 10.1159/000529187

**Published:** 2023-03-30

**Authors:** Tanja van Essen, Norani H. Gangaram-Panday, Willem van Weteringen, Tom G. Goos, Irwin K.M. Reiss, Rogier C.J. de Jonge

**Affiliations:** ^a^Division of Neonatology, Department of Pediatrics, Erasmus MC Sophia Children's Hospital, University Medical Center Rotterdam, Rotterdam, The Netherlands; ^b^Department of Biomechanical Engineering, Delft University of Technology, Delft, The Netherlands; ^c^Pediatric Intensive Care Unit, Department of Pediatrics and Pediatric Surgery, Erasmus MC Sophia Children's Hospital, University Medical Center Rotterdam, Rotterdam, The Netherlands

**Keywords:** Microcirculation, Transcutaneous, Blood gas, Neonate

## Abstract

**Introduction:**

Transcutaneous blood gas monitoring allows for continuous non-invasive evaluation of carbon dioxide and oxygen levels. Its use is limited as its accuracy is dependent on several factors. We aimed to identify the most influential factors to increase usability and aid in the interpretation of transcutaneous blood gas monitoring.

**Methods:**

In this retrospective cohort study, transcutaneous blood gas measurements were paired to arterial blood gas withdrawals in neonates admitted to the neonatal intensive care unit. The effects of patient-related, microcirculatory, macrocirculatory, respiratory, and sensor-related factors on the difference between transcutaneously and arterially measured carbon dioxide and oxygen values (ΔPCO<sub>2</sub> and ΔPO<sub>2</sub>) were evaluated using marginal models.

**Results:**

A total of 1,578 measurement pairs from 204 infants with a median [interquartile range] gestational age of 27<sup>3</sup>/<sub>7</sub> [26<sup>1</sup>/<sub>7</sub>–31<sup>3</sup>/<sub>7</sub>] weeks were included. ΔPCO<sub>2</sub> was significantly associated with the postnatal age, arterial systolic blood pressure, body temperature, arterial partial pressure of oxygen (PaO<sub>2</sub>), and sensor temperature. ΔPO<sub>2</sub> was, with the exception of PaO<sub>2</sub>, additionally associated with gestational age, birth weight Z-score, heating power, arterial partial pressure of carbon dioxide, and interactions between sepsis and body temperature and sepsis and the fraction of inspired oxygen.

**Conclusion:**

The reliability of transcutaneous blood gas measurements is affected by several clinical factors. Caution is recommended when interpreting transcutaneous blood gas values with an increasing postnatal age due to skin maturation, lower arterial systolic blood pressures, and for transcutaneously measured oxygen values in the case of critical illness.

## Introduction

Transcutaneous blood gas monitoring provides non-invasive continuous measurements of the partial pressures of carbon dioxide (tcPCO_2_) and oxygen (tcPO_2_), and is mostly used in neonatal intensive care [1, 2]. Monitoring of tcPCO_2_ is an attractive alternative to capnography, which adds dead space ventilation [3]. However, the accuracy of transcutaneous blood gas measurements is often questioned and remains a topic of scientific investigation [4, 5, 6]. This is partially caused by the often extreme inaccuracy of tcPO_2_, which can largely be explained by the mechanism behind transcutaneous blood gas monitoring. Transcutaneous sensors locally heat the skin with the primary goal of inducing local vasodilatation, thereby “arterializing” the skin. The considerable increase in local blood flow equilibrates skin carbon dioxide (CO_2_) and oxygen (O_2_) levels to arterial values, reducing the contribution of local CO_2_ production and O_2_ consumption [7]. It has been shown that changes in local skin perfusion caused by changes in temperature and blood pressure can still have a notable effect on blood gas diffusion, and with it sensor accuracy [6, 8, 9]. The fact that O_2_ diffuses 20 times slower than CO_2_ makes it more prone to these changes, despite adequate heating of the skin [10].

The difficulty in identifying the cause of inaccuracy often leads to technical blame, which is understandable considering the effects that defective sensor membranes and aging electrolyte solutions can have. However, inaccuracy of transcutaneous blood gas monitoring can, to a large part, be attributed to patient-related factors that affect the diffusion of blood gases in the skin [4, 11, 12]. Determination of factors that affect measurement accuracy could be of considerable value for improving the clinical usability and increasing the use of transcutaneous blood gas monitoring. Therefore, the aim of this study was to identify patient-related, microcirculatory, macrocirculatory, respiratory, and device-related factors affecting the accuracy of transcutaneous monitoring of CO_2_ and O_2_ in the neonatal intensive care unit (NICU).

## Methods

### Study Population

A retrospective cohort study was conducted. Data on transcutaneously and arterially measured CO_2_ and O_2_ values were collected between November 2015 and August 2018 at the level III NICU of Erasmus MC Sophia Children's Hospital (Rotterdam, The Netherlands). All infants at the NICU with an arterial line, on invasive ventilation and transcutaneous blood gas monitoring, were eligible for inclusion. The Local Medical Ethical Review Board waived approval for this study.

### Transcutaneous Blood Gas Measurements

Measurements of tcPCO_2_ and tcPO_2_ were performed with an Oxivent^TM^ Sensor (software versions 01.57–01.58; Sentec AG, Therwil, Switzerland) and Sentec Digital Monitor (software versions 08.00.0–08.02.1; Sentec AG, Therwil, Switzerland). According to local protocol, sensor temperatures and site times were set to 42°C/2 h for neonates ≤25 weeks of gestational age (GA) and to 43°C/3 h for neonates >25 weeks of GA. After elapsing of the site time, the sensor temperature was automatically lowered to 39°C to prevent skin burns. TcPCO_2_ was real-time calculated from a pH measurement using a formula which corrects for sensor temperature. Contrary to previous generations of transcutaneous oxygen sensors, the applied oxygen measurement was based on fluorescence quenching, which does not consume oxygen. TcPO_2_ was per sensor factory-calibrated to a range of temperatures. Sensors were calibrated against a reference gas mixture. TcPCO_2_ calibration was mandatory after the site time elapsed, and tcPO_2_ calibrated automatically every 24 h during a tcPCO_2_ calibration. In vivo calibrations to blood gas samples or custom measurement offsets were not applied.

### Sample Selection

Arterial blood gas withdrawal was performed on clinical indication. For data pairing, the exact timing of arterial blood gas withdrawal was identified from the visible disruption of the arterial blood pressure curve [11]. Data pairs were excluded when recorded after an elapsed site time, within a ten-minute stabilization window following a calibration or during therapeutic hypothermia.

### Evaluated Variables

#### Factors Related to the Patient

General patient factors taken into account were GA, gender, and birth weight of the infant. Birth weight was corrected for GA and presented as a Z-score [13]. Postnatal age was defined as the number of days between birth and the moment of blood sampling, and was used as a proxy for skin maturation.

#### Factors Related to Macrocirculation

The systolic blood pressure after blood sampling was included as an indicator of the arterial blood pressure. Heart rate was primarily derived from electrocardiography (ECG). In the absence of ECG, the heart rate was obtained from pulse oximetry.

#### Factors Related to the Microcirculation

Conditions with an effect on the cutaneous circulation, such as sepsis, necrotizing enterocolitis (NEC), and body temperature, were evaluated. Data pairs were classified as septic or non-septic based on a blood culture. Samples were marked as septic from 1 day before a positive blood culture until the end of antibiotic treatment. Sample pairs were classified as “during NEC” from 1 day before until 1 day after surgery for NEC with Bell stages II to III.

#### Respiratory Factors

The mode of ventilation (high-frequency oscillatory [HFO] or other invasive ventilation) and fraction of inspired oxygen (FiO_2_) were collected. Additionally, the effects of the arterial partial pressures of CO_2_ (PaCO_2_) and O_2_ (PaO_2_) were evaluated.

#### Sensor-Related Factors

The heating power (mW) of the sensor was used as a proxy for cutaneous blood flow, as the total power needed to maintain a stable sensor temperature is strongly influenced by the local cutaneous blood flow [14]. Additionally, the set sensor temperature was included.

### Data Acquisition

TcPCO_2_, tcPO_2_, heating power levels, and the sensor temperature were logged at 1 Hz (Raspberry Pi 2 or 3 model B; Raspberry Pi Foundation, UK). Standard of care patient monitoring data including heart rate (ECG or pulse oximetry), invasive arterial blood pressure, and body temperature (Dräger M540; Drägerwerk AG & Co. KGaA, Lübeck, Germany; Masimo SET, Irvine, CA, USA) was logged at 1 Hz. High-frequency (100 Hz) blood pressure tracings were recorded as standard of care. Demographic data, data on ventilation methods, FiO_2_, blood cultures, antibiotic treatment, and laboratory data were collected from the electronic patient records (PDMS; Picis Clinical Solutions, Wakefield, MA, USA, and HiX version 6.1; Chipsoft, Amsterdam, The Netherlands).

### Statistical Analysis

Categorical variables are presented as number (%) and continuous variables as median (interquartile range). Agreement between transcutaneous blood gas measurements and arterial blood gas samples was calculated according to Bland and Altman, accounting for multiple measurements per patient [15]. To identify factors associated with the difference between arterial and transcutaneous blood gas values (transcutaneous − arterial blood gas values; ΔPCO_2_ and ΔPO_2_), marginal models were used. The described variables were included in the models, with PaO_2_ only in the CO_2_ model and PaCO_2_ only in the O_2_ model. To allow for non-linearity in the relation between continuous explanatory variables and the outcome, splines were evaluated with boundary knots at the 5th and 95th percentile. The following interactions were considered and added to the model when significant: FiO_2_ and ventilation mode; FiO_2_ and sepsis state; arterial systolic blood pressure and sepsis state; body temperature and sepsis state. To account for the within-subject correlations of repeated measures, a compound symmetry covariance matrix was applied in the CO_2_ model and a continuous first-order autoregressive covariance matrix in the O_2_ model. Additionally, the relation between ΔPO_2_ and ΔPCO_2_ was evaluated using a marginal model, adjusting for all significant variables from the CO_2_ model. A two-sided *p* value of <0.05 was considered statistically significant. All analyses were performed using R statistical software (version 4.1.1; The *R* Foundation for Statistical Computing, Vienna, Austria), using the nlme package [16].

## Results

A total of 1,897 data pairs were obtained from 214 patients during the study period. After exclusion of pairs measured at a sensor temperature of 39°C (*n* = 58), during therapeutic hypothermia (*n* = 60) and surrounding a calibration (*n* = 201), 1,578 samples from 204 patients were included for analyses. Table 1 summarizes demographic and clinical data for patients and samples. The Bland-Altman analysis showed a bias and 95% limits of agreement of 4.5 (−14.5–23.4) mm Hg for CO_2_ and −16.1 (−63.1–30.9) mm Hg for O_2_.

### ΔPCO_2_

**Fig. 1:**
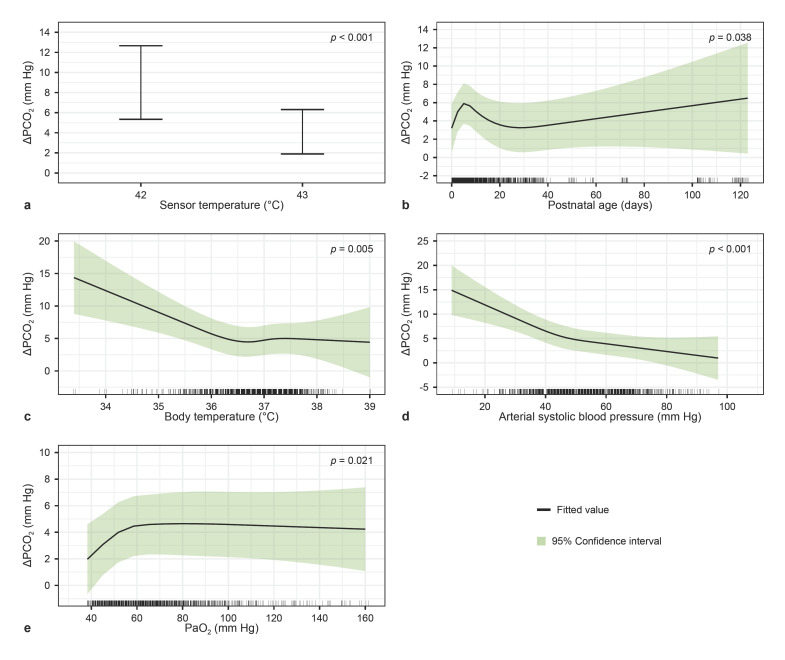
Effect plots of the CO_2_ model, describing the relation between multiple factors and the observed difference between tcPCO_2_ and PaCO_2_ (ΔPCO_2_). The bold lines represent the estimates, and shaded areas represent the 95% confidence intervals. Independent variables of significant influence in the model. **a** Sensor temperature (42°C/43°C). **b** Postnatal age (days). **c** Body temperature (°C). **d** Arterial systolic blood pressure (mm Hg). **e** PaO_2_ (mm Hg); for PaCO_2_, the *X*-axis is truncated at the 1st and 99th percentile to improve readability. TcPCO_2_, transcutaneous carbon dioxide levels; PaCO_2_, arterial partial pressure of carbon dioxide; ΔPCO_2_, difference between transcutaneous and arterial carbon dioxide levels; PaO_2_, arterial partial pressure of oxygen.

None of the interactions significantly improved the model and were therefore not included. The ΔPCO_2_ was significantly influenced by postnatal age, arterial systolic blood pressure, body temperature, PaO_2_, and sensor temperature (Table 2). The relation between significant factors and ΔPCO_2_ is presented in Figure 1 as the estimate with the 95% confidence interval (CI). A sensor temperature of 43°C resulted in a significantly smaller ΔPCO_2_ when compared to 42°C. A body temperature below 36.5°C resulted in an increase in ΔPCO_2_. ΔPCO_2_ increased rapidly in the first week after birth. Lower arterial systolic blood pressures resulted in a larger ΔPCO_2._ The full model output is shown in online supplementary Table 1 (see www.karger.com/doi/10.1159/000529187 for all online suppl. material).

### ΔPO_2_

**Fig. 2:**
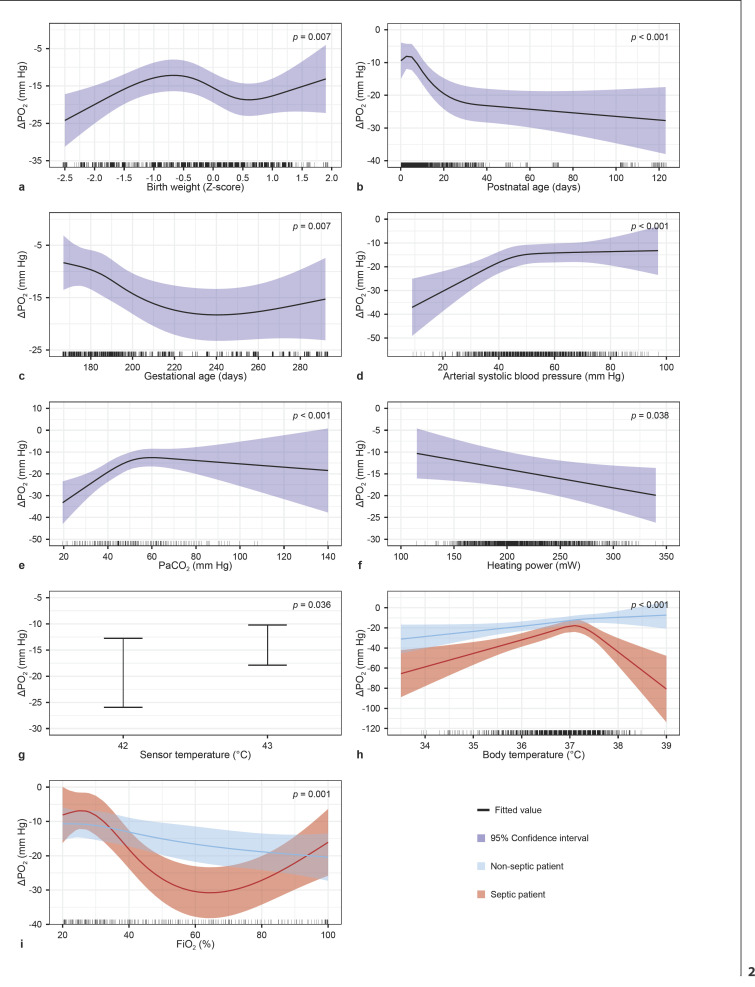
Effect plots of the O_2_ model, describing the relation between multiple factors and the observed difference between tcPO_2_ and PaO_2_ (ΔPO_2_). The bold lines represent the estimates, and shaded areas represent the 95% confidence intervals. Independent variables with a significant relation included in the model. **a** Birth weight presented as Z-score. **b** Postnatal age (days). **c** Gestational age (days). **d** Arterial systolic blood pressure (mm Hg). **e** PaCO_2_ (mm Hg). **f** Heating power (mW). **g** Sensor temperature (°C). **h** Interaction between body temperature (°C) and sepsis (yes/no). **i** Interaction between FiO_2_ (%) and sepsis (yes/no). TcPO_2_, transcutaneous oxygen levels; PaO_2_, arterial partial pressure of oxygen; ΔPO_2_, difference between transcutaneous and arterial oxygen levels; PaCO_2_, arterial partial pressure of carbon dioxide; FiO_2_, fraction of inspired oxygen.

The ΔPO_2_ was significantly influenced by GA, birth weight Z-score, postnatal age, arterial systolic blood pressure, body temperature, FiO_2_, PaCO_2_, heating power, sensor temperature. Interactions were significant between FiO_2_ and sepsis state and between body temperature and sepsis state (Table 2). The effect plots of the estimates and 95% CI are shown in Figure 2. The ΔPO_2_ increased mostly within the first 20 days after birth. ΔPO_2_ increased for both arterial systolic blood pressures below 45 mm Hg and PaCO_2_ values below 50 mm Hg. A temperature of 43°C resulted in a significantly smaller ΔPO_2_. An increase in heating power showed an increase in ΔPO_2_. In addition, the ΔPO_2_ decreased with an increasing body temperature. For septic infants, the ΔPO_2_ was larger than for non-septic infants and increased substantially for body temperatures above 37°C. The effect of FiO_2_ on ΔPO_2_ differed between septic and non-septic infants. The full model output is shown in online supplementary Table 2.

### Relation between ΔPCO_2_ and ΔPO_2_

**Fig. 3:**
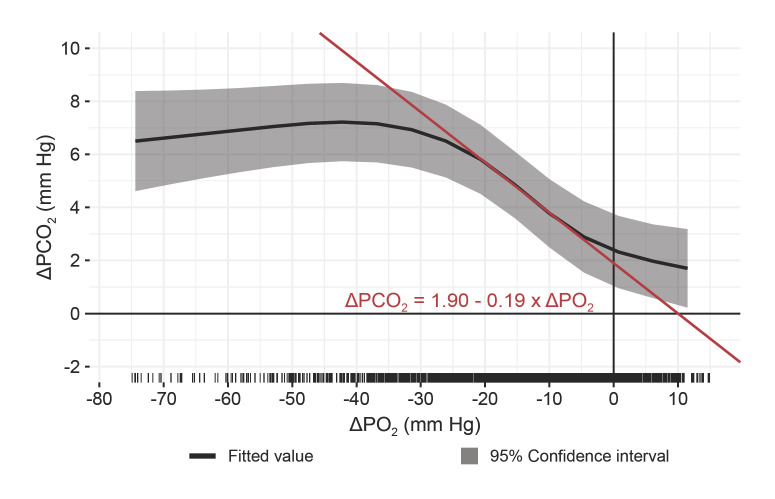
Effect plot describing the relation between ΔPCO_2_ and ΔPO_2_. The bold line represents the estimate, and the shaded area represents the 95% confidence interval. The steep incline (red line) in ΔPCO_2_ for an increase in ΔPO_2_ is described by ΔPCO_2_ = 1.90–0.19 × ΔPO_2_. ΔPCO_2_, difference between transcutaneous and arterial carbon dioxide levels; ΔPO_2_, difference between transcutaneous and arterial oxygen levels.

Figure 3 illustrates the relation between ΔPCO_2_ and ΔPO_2_, presented as estimate and 95% CI. For a ΔPO_2_ between −5 mm Hg and −25 mm Hg, an increase of 1 mm Hg resulted in a 0.19 mm Hg increase in ΔPCO_2_.

## Discussion

This study identified various factors related to the patient, microcirculation, macrocirculation, and sensor that affect agreement between transcutaneous blood gas values and arterial reference samples. The ΔPCO_2_ was mainly affected by low arterial systolic blood pressure, body temperature, and sensor temperature, as well as postnatal age. In addition to these factors, ΔPO_2_ was affected by GA, birth weight Z-score, PaCO_2_, heating power, and sepsis in relation to body temperature and FiO_2_ levels.

The ΔPCO_2_ and ΔPO_2_ show an increase with both an increasing postnatal age and GA, which is a known effect of skin development on transcutaneous blood gas measurements [17]. Intrauterine development of the stratum corneum lasts until approximately 34 weeks of gestation, during which the distance between skin capillaries and the skin surface increases [18]. Postnatally, the skin keratinizes in 2–3 weeks [18]. Both processes reduce the diffusion capacity of the skin for O_2_ and to a lesser extent for CO_2_.

Transcutaneous blood gases are often measured in hemodynamically instable neonates. Arterialization of the skin reduces vascular autoregulation, making cutaneous flow primarily blood pressure dependent [19, 20]. Our study shows that an arterial systolic blood pressure below approximately 50 mm Hg decreases tcPCO_2_ and tcPO_2_ accuracy. Previous literature described a systolic blood pressure below 30 mm Hg to influence the reliability of tcPO_2_ measurements [9, 21]. Heart rate was included as an indicator of cardiac output, as in neonates changes in cardiac output are largely dependent on changes in heart rate [22]. The fact that heart rate is not significantly associated with ΔPCO_2_ or ΔPO_2_ can be explained by values in the normal range and inclusion of blood pressure in the models. Literature shows that in adults only a severely reduced cardiac output (e.g., resuscitation and severe shock) affects transcutaneous blood gas measurements [23].

The presence of sepsis had no effect on ΔPCO_2_. This suggests that during sepsis cutaneous flow is sufficient to provide accurate tcPCO_2_ values, to which the high diffusion speed of CO_2_ attributes [10]. However, tcPO_2_ levels were consistently lower in septic infants, in particular when accompanied by an elevated body temperature. Unfortunately, a limited number of samples with a body temperature above 38°C were available. Future studies should investigate the effect of sepsis.

Under physiological pulmonary and microcirculatory conditions, an increase in FiO_2_ leads to an increase in PaO_2_ and tcPO_2_. The increase in tcPO_2_ levels found in this study was limited, possibly indicating a maximum diffusion capacity of the skin [19]. This effect is more pronounced in septic infants, which can be attributed to a reduced peripheral circulation [11, 12]. The significant effects of PaO_2_ and PaCO_2_ could be a consequence of changes in regional blood flow, invoked by changes in tissue O_2_, CO_2_, and pH that alter local metabolic activity [24].

The interaction between the heated sensor and the microcirculation is expressed in several parameters. The effect of sensor temperature has been investigated extensively [8, 25], yet the chosen temperature differs strongly per hospital, country, and severity of prematurity. It is often still historically motivated by a fear for skin burns, despite the improvement that closed-loop temperature control nowadays provides. Sensor temperatures up to 44°C yield a higher accuracy and are likely to reduce the influence of several factors. In this study, a significant effect of heating power on ΔPO_2_ was found. A higher heating power was related to a larger ΔPO_2_, and this could be attributed to a combined effect of changes in blood flow and other factors, such as skin thickness. Analysis of continuous heating power data and the inclusion of blood flow measurements may provide more insight into this phenomenon.

An interesting finding of this study was the increase of ΔPO_2_ with an increase in ΔPCO_2_. Although the diffusion gradients in unheated skin have opposing directions, in heated skin they are directed outward and to a different degree affected by the same factors. This suggests a common dependency on the blood flow under the sensor.

Correct use of transcutaneous blood gas monitors, including frequent calibrations, leak-free sensor fixation, and timely renewal of the sensor membrane, is paramount for obtaining valid measurements. Sensor location and the presence of edema at the measurement site could influence accuracy, but were not recorded in this study. Sensor calibrations were mandatory for measurement continuation. The NICU staff received frequent and extensive training on sensor use and quality assessment, limiting the influence of these sensor-related factors. For continuous variables, such as the heart rate, only a single measurement value during arterial blood gas withdrawal was included in the analysis. The effect of fluctuation of these variables could therefore not be evaluated. In addition, the fluorescence quenching technique for measurement of tcPO_2_ does not influence diffusion of oxygen toward the sensor. Results should be interpreted with care when study results are compared to measurements obtained with the traditionally used Clark electrode.

Clinical interpretation of transcutaneous blood gas measurements is challenging due to the many factors simultaneously influencing accuracy. Arterial blood gas measurement remains the golden standard for intermittent evaluation of CO_2_ and O_2_ levels in infants. When used correctly, transcutaneous blood gas measurements provide a valuable continuous evaluation of blood gases in neonates. The complexity of using and maintaining transcutaneous sensors is the main reason that the convenience of using pulse oximetry is often preferred despite their inaccurate estimation of oxygenation. Besides the use of transcutaneous blood gases for respiratory monitoring, there is an increasing interest in its value as an indicator of tissue perfusion and hemodynamic failure, such as cardiac decompensation, shock, sepsis, and clinical outcome [12, 26]. This study identified factors that affect accuracy and reliability of transcutaneous blood gas monitoring in neonates to improve clinical usability. Further research needs to be conducted in order to prove their value for determining accuracy in various clinical settings.

## Conclusion

Several clinical factors have been identified that influence the agreement between arterial and transcutaneous blood gas values.

- Maturation of the skin reduces accuracy of both tcPO_2_ and tcPCO_2_ following the first period after birth.
- An arterial systolic blood pressure below approximately 50 mm Hg significantly impairs transcutaneous blood gas measurements.
- Hypocapnia leads to an inaccuracy of tcPO_2_ measurements.
- Caution is recommended with critical illness, as tcPO_2_ may deviate from arterial values.

## Statement of Ethics

The Medical Ethical Review Board of the Erasmus MC, Rotterdam, The Netherlands, waived approval for this study (“Medical Research in Human Subjects Act does not apply to this research proposal”; MEC-2018-1682).

## Conflict of Interest Statement

The authors have no conflicts of interest to declare.

## Funding Sources

This study was partly funded by Sentec AG. Sentec AG had no role in the design and conduct of the study, data analysis, interpretation of results, or writing of the manuscript.

## Author Contributions

Tanja van Essen, Norani H. Gangaram-Panday, Willem van Weteringen, Tom G. Goos, Irwin K.M. Reiss, and Rogier C.J. de Jonge conceptualized the study. Tanja van Essen, Norani H. Gangaram-Panday, and Willem van Weteringen collected the data and wrote the first draft of the manuscript. Tanja van Essen and Norani H. Gangaram-Panday analyzed the data. All authors have provided valuable input on the writing of the final manuscript.

## Data Availability Statement

All data generated or analyzed during this study are included in this article and its online supplementary material. Further inquiries can be directed to the corresponding author.

## Supplementary Material

Supplementary data

## Figures and Tables

**Table 1:** Demographics and clinical data

	*N*	*n* (%)
*Patients (n = 204)*		
Female gender	204	86 (42)
Gestational age, weeks	204	27^3^/_7_ [26^1^/_7_−31^3^/_7_]
Birth weight, g	204	960 [749–1,600]
Birth weight, Z-score	204	0.0 [–0.9–0.6]
Caesarean section	204	130 (64)
Apgar		
Min 1	197	5 [3–7]
Min 5	197	7 [6–8]
Min 10	176	8 [8–9]
Umbilical cord pH	153	7.30 [7.21–7.35]
Multiple births	204	34 (17)
Admission survival	204	146 (72)
Sepsis during admission	204	85 (44)
NEC	204	49 (24)
Surgery for NEC^1^		40 (82)

*Samples (n = 1,578)*		
Postmenstrual age at sample, weeks	1,578	28^6^/_7_ [27^1^/_7_−33^6^/_7_]
Postnatal age, days	1,578	6 [3–12]
Sepsis state		
Sepsis	1,578	324 (21)
No sepsis		1,254 (79)
Sensor temperature		
43°C	1,578	1,430 (91)
42°C		148 (9)
Ventilation mode^2^		
Invasive	1,511	640 (42)
HFO		871 (58)

Values are presented as median [interquartile range] or *n* (%). NEC, necrotizing enterocolitis; HFO, high-frequency oscillatory.

1 One infant died before surgical strategy could be determined.

2 Lost data due to change in patient record system.

**Table 2:** Marginal *p* values of ΔPCO_2_ and ΔPO_2_ models

ΔPCO_2_	Marginal *p* value	ΔPO_2_	Marginal *p* value
Intercept	**0.032**	Intercept	**0.003**
Gender (female)	0.856	Gender (female)	0.798
Gestational age (days)	0.444	Gestational age (days)	**0.007**
Birth weight (Z-score)	0.415	Birth weight (Z-score)	**0.007**
Postnatal age (days)	**0.038**	Postnatal age (days)	**<0.001**
Arterial systolic blood pressure (mm Hg)	**<0.001**	Arterial systolic blood pressure (mm Hg)	**<0.001**
Heart rate (bpm)	0.139	Heart rate (bpm)	0.148
Sepsis (yes)	0.270	Sepsis (yes)	0.127
NEC (no)	0.114	NEC (no)	0.920
Body temperature (°C)	**0.005**	Body temperature (°C)	**<0.001**
FiO_2_ at sample (%)	0.071	FiO_2_ at sample (%)	**<0.001**
Ventilation mode (HFO)	0.812	Ventilation mode (HFO)	0.791
PaO_2_ (mm Hg)	**0.021**	PaCO_2_ (mm Hg)	**<0.001**
Heating power (mW)	0.936	Heating power (mW)	**0.038**
Sensor temperature (42°C)	**<0.001**	Sensor temperature (42°C)	**0.036**
		**Interactions**	
		FiO_2_ at sample (%) and sepsis (yes)	**0.001**
		Body temperature (°C) and sepsis (yes)	**<0.001**

ΔPCO_2_, difference between transcutaneous and arterial carbon dioxide levels; ΔPO_2_, difference between transcutaneous and arterial oxygen levels; NEC, necrotizing enterocolitis; FiO_2_, fraction of inspired oxygen; HFO, high-frequency oscillatory; PaCO_2_, arterial partial pressure of carbon dioxide; PaO_2_, arterial partial pressure of oxygen.
