# Supplementary material for: Improving the Clinical Interpretation of Transcutaneous Carbon Dioxide and Oxygen Measurements in the Neonatal Intensive Care Unit
Source: Neonatology. 2023 Mar 30;120(3):308–16. doi: 10.1159/000529187 (PMC10389786; doi:10.1159/000529187)
Supplement: Supplementary file 1 — Supplementary data [file neo-0120-0308-s01.docx]

**Supplemental**

| **Supplemental Table 1** Full model output for ΔPCO_2_ | | | | | | |
| --- | --- | --- | --- | --- | --- | --- |
|  | Splines | Beta | SE | t-value | P-value | Marginal P-value |
| Intercept | 1 | 7.89 | 3.67 | 2.15 | 0.032 | **0.032** |
| Gender (Female) | 1 | -0.18 | 0.98 | -0.18 | 0.856 | 0.856 |
| Gestational age (days) | 1 | -2.89 | 2.35 | -1.23 | 0.219 | 0.444 |
| Gestational age (days) | 2 | -1.58 | 2.89 | -0.55 | 0.586 |  |
| Gestational age (days) | 3 | -1.98 | 1.88 | -1.05 | 0.293 |  |
| Birth weight (Z-score) | 1 | 2.28 | 1.63 | 1.40 | 0.162 | 0.415 |
| Birth weight (Z-score) | 2 | 3.63 | 3.11 | 1.17 | 0.244 |  |
| Birth weight (Z-score) | 3 | 0.73 | 1.39 | 0.53 | 0.598 |  |
| Postnatal age (days) | 1 | -2.08 | 1.36 | -1.53 | 0.126 | **0.038** |
| Postnatal age (days) | 2 | 1.90 | 1.90 | 1.00 | 0.318 |  |
| Postnatal age (days) | 3 | -1.48 | 1.15 | -1.29 | 0.197 |  |
| Systolic blood pressure (mm Hg) | 1 | -3.23 | 1.01 | -3.19 | <0.001 | **<0.001** |
| Systolic blood pressure (mm Hg) | 2 | -7.89 | 1.46 | -5.40 | <0.001 |  |
| Systolic blood pressure (mm Hg) | 3 | -3.84 | 0.97 | -3.97 | <0.001 |  |
| Heart rate (bpm) | 1 | -1.24 | 1.04 | -1.19 | 0.233 | 0.139 |
| Heart rate (bpm) | 2 | -2.92 | 1.83 | -1.60 | 0.110 |  |
| Heart rate (bpm) | 3 | 0.27 | 0.99 | 0.27 | 0.785 |  |
| Sepsis (yes) | 1 | -1.04 | 0.94 | -1.10 | 0.270 | 0.270 |
| NEC (no) | 1 | -1.84 | 1.17 | -1.58 | 0.114 | 0.114 |
| Body temperature (°C) | 1 | -0.58 | 0.90 | -0.65 | 0.518 | **0.005** |
| Body temperature (°C) | 2 | -4.15 | 1.28 | -3.24 | 0.001 |  |
| Body temperature (°C) | 3 | -0.28 | 0.85 | -0.33 | 0.739 |  |
| FiO_2_ at sample (%) | 1 | 1.60 | 1.38 | 1.16 | 0.247 | 0.071 |
| FiO_2_ at sample (%) | 2 | 4.13 | 1.60 | 2.58 | 0.010 |  |
| FiO_2_ at sample (%) | 3 | 1.79 | 1.09 | 1.64 | 0.102 |  |
| Ventilation mode (HFO) | 1 | 0.15 | 0.64 | 0.24 | 0.812 | 0.812 |
| PaO_2_ (mm Hg) | 1 | 1.58 | 0.85 | 1.86 | 0.063 | **0.021** |
| PaO_2_ (mm Hg) | 2 | 4.11 | 1.34 | 3.08 | 0.002 |  |
| PaO_2_ (mm Hg) | 3 | 1.18 | 0.76 | 1.55 | 0.121 |  |
| Heating power (mW) | 1 | 0.00 | 0.01 | -0.08 | 0.936 | 0.936 |
| Sensor temperature (42 °C) | 1 | 4.92 | 1.42 | 3.46 | <0.001 | **<0.001** |

Systolic blood pressure: arterial systolic blood pressure, SE: standard error, NEC: necrotizing enterocolitis, FiO_2_: fraction of inspired oxygen, HFO: high-frequency oscillatory, PaO_2_: arterial partial pressure of oxygen.

| **Supplemental Table 2** Full model output for ΔPO_2_ | | | | | | |
| --- | --- | --- | --- | --- | --- | --- |
|  | Splines | Beta | SE | t-value | P-value | Marginal P-value |
| Intercept | 1 | -24.36 | 8.11 | -3.00 | <0.001 | **0.003** |
| Gender (Female) | 1 | 0.40 | 1.57 | 0.26 | 0.798 | 0.798 |
| Gestational age (days) | 1 | -11.87 | 3.94 | -3.02 | 0.003 | **0.007** |
| Gestational age (days) | 2 | -10.82 | 4.63 | -2.34 | 0.020 |  |
| Gestational age (days) | 3 | -6.84 | 3.30 | -2.07 | 0.039 |  |
| Birth weight (Z-score) | 1 | -4.79 | 2.65 | -1.81 | 0.071 | **0.007** |
| Birth weight (Z-score) | 2 | 10.68 | 4.82 | 2.21 | 0.027 |  |
| Birth weight (Z-score) | 3 | -3.81 | 2.31 | -1.65 | 0.099 |  |
| Postnatal age (days) | 1 | -13.74 | 2.90 | -4.74 | <0.001 | **<0.001** |
| Postnatal age (days) | 2 | -11.62 | 4.43 | -2.62 | 0.009 |  |
| Postnatal age (days) | 3 | -15.11 | 2.49 | -6.07 | <0.001 |  |
| Systolic blood pressure (mm Hg) | 1 | 5.66 | 2.46 | 2.30 | 0.021 | **<0.001** |
| Systolic blood pressure (mm Hg) | 2 | 14.73 | 3.53 | 4.17 | <0.001 |  |
| Systolic blood pressure (mm Hg) | 3 | 4.65 | 2.30 | 2.02 | 0.044 |  |
| Heart rate (bpm) | 1 | -3.06 | 2.51 | -1.22 | 0.223 | 0.148 |
| Heart rate (bpm) | 2 | -1.67 | 4.41 | -0.38 | 0.706 |  |
| Heart rate (bpm) | 3 | -5.08 | 2.37 | -2.14 | 0.032 |  |
| Sepsis (yes) | 1 | 7.15 | 4.68 | 1.53 | 0.127 | 0.127 |
| NEC (no) | 1 | -0.30 | 2.94 | -0.10 | 0.920 | 0.920 |
| Body temperature (°C) | 1 | 17.67 | 4.78 | 3.70 | <0.001 | **<0.001** |
| Body temperature (°C) | 2 | 20.43 | 5.90 | 3.47 | 0.001 |  |
| Body temperature (°C) | 3 | -2.74 | 4.59 | -0.60 | 0.550 |  |
| FiO_2_ at sample (%) | 1 | -36.32 | 6.71 | -5.41 | <0.001 | **<0.001** |
| FiO_2_ at sample (%) | 2 | -17.94 | 7.50 | -2.39 | 0.017 |  |
| FiO_2_ at sample (%) | 3 | -9.55 | 4.91 | -1.95 | 0.052 |  |
| Ventilation mode (HFO) | 1 | 0.39 | 1.47 | 0.27 | 0.791 | 0.791 |
| PaCO_2_ (mm Hg) | 1 | 9.57 | 2.05 | 4.67 | <0.001 | **<0.001** |
| PaCO_2_ (mm Hg) | 2 | 17.28 | 3.32 | 5.21 | <0.001 |  |
| PaCO_2_ (mm Hg) | 3 | 7.57 | 1.83 | 4.15 | <0.001 |  |
| Heating power (mW) | 1 | -0.04 | 0.02 | -2.08 | 0.038 | **0.038** |
| Sensor temperature (42 °C) | 1 | -5.30 | 2.52 | -2.10 | 0.036 | **0.036** |
| **Interactions** |  |  |  |  |  |  |
| FiO_2_ at sample (%)  and sepsis (yes) | 1 | 29.78 | 7.61 | 3.91 | <0.001 | **0.001** |
| FiO_2_ at sample (%)  and sepsis (yes) | 2 | 9.58 | 8.22 | 1.17 | 0.244 |  |
| FiO_2_ at sample (%)  and sepsis (yes) | 3 | -0.15 | 5.68 | -0.03 | 0.979 |  |
| Body temperature (°C)  and sepsis (yes) | 1 | -10.52 | 5.28 | -1.99 | 0.047 | **<0.001** |
| Body temperature (°C)  and sepsis (yes) | 2 | -7.35 | 6.62 | -1.11 | 0.267 |  |
| Body temperature (°C)  and sepsis (yes) | 3 | 10.23 | 4.90 | 2.09 | 0.037 |  |

Systolic blood pressure: arterial systolic blood pressure, SE: standard error, NEC: necrotizing enterocolitis, FiO_2_: fraction of inspired oxygen, HFO: high-frequency oscillatory, PaCO_2_: arterial partial pressure of carbon dioxide.
